# EHMTI-0091. Physiotherapy interventions for headaches: a systematic review and meta-analysis

**DOI:** 10.1186/1129-2377-15-S1-D38

**Published:** 2014-09-18

**Authors:** K Luedtke, A May

**Affiliations:** 1 Institute of Systems Neurosciences, University Medical Center Hamburg-Eppendorf, Hamburg, Germany

## Background

Headaches are a common condition with high socioeconomic impact.

Guidelines recommend medication but rarely include physiotherapy. However, patients report pain relief from exercises, mobilisation and massage. A systematic review with a literature search up to 11/2002 concluded that physical treatments may be effective but that further research might change this result.

## Aim

To evaluate the current level of evidence for physiotherapy on headache symptoms.

## Methods

Search strategy with pre-defined key terms conducted in MEDLINE, CENTRAL, PeDRO, reference lists of retrieved articles, and journal contents. Controlled trials, employing physiotherapy interventions for the reduction of headaches published in any language between 11/2002 and 04/2014 were included. Studies using chiropractic, osteopathic or acupuncture techniques were excluded. Quality was evaluated using the Cochrane risk of bias tool.

## Results

Twenty-two trials were eligible, 19 reported outcome measures allowing combination in a meta-analysis. Physiotherapy included mobilisation, exercises, relaxation, massage, and physiotherapy as part of a multidisciplinary intervention. Headache types included migraine, TTH, CGH and mixed/undefined headache populations. A meta-analysis for pain reduction found a pooled effect size of -9.97 on a 0-100 VAS (95%CI -18.55; -1.38). Frequency was reduced by -2.78 days/months (95%CI -4.69; -0.87), duration by -7.52 hours/attack (95%CI -10.49; -4.55). Sub-analyses indicated effectiveness for the reduction of pain intensity, frequency and duration of migraine, TTH, CGH and mixed/undefined headache populations.

## Conclusions

There is a high level of evidence that physiotherapy is effective for headache reduction. Physiotherapy is low-cost has no side-effects and potentially reduces medication use and work absenteeism.

No conflict of interest.

